# Cyclone-induced hematological emergencies: preparedness and response from a hematologist’s viewpoint

**DOI:** 10.1097/MS9.0000000000003475

**Published:** 2025-06-12

**Authors:** Emmanuel Ifeanyi Obeagu

**Affiliations:** a Department of Biomedical and Laboratory Science, Africa University, MUutare, Zimbabwe

**Keywords:** blood disorders, cyclone disasters, disaster preparedness, hematological emergencies, transfusion management

## Abstract

Cyclone disasters pose significant health challenges, including severe hematological emergencies resulting from trauma, infections, and systemic inflammatory responses. Injuries sustained during cyclones can lead to trauma-induced coagulopathy, acute anemia, and disseminated intravascular coagulation (DIC), requiring immediate intervention. Additionally, post-disaster conditions such as malnutrition, dehydration, and poor sanitation contribute to increased susceptibility to infections, further exacerbating blood-related disorders. Hematologists play a vital role in diagnosing and managing these complications through rapid hematological assessments, transfusion support, and coagulation monitoring. Disaster preparedness is crucial in mitigating the impact of cyclone-induced hematological disorders. Ensuring an adequate blood supply, establishing emergency transfusion protocols, and collaborating with disaster response teams are essential pre-disaster strategies. Post-cyclone responses require hematologists to triage affected individuals, manage thromboembolic risks, and monitor sepsis-related hematological dysfunctions. Long-term rehabilitation efforts should focus on nutritional support, anemia management, and continuous surveillance of blood disorders in displaced populations to prevent chronic hematological complications.

## Introduction

Cyclone disasters are among the most devastating natural calamities, causing widespread destruction, injuries, and fatalities. In addition to infrastructural damage and displacement, these disasters significantly impact public health, leading to an increased burden of trauma, infections, and systemic disorders. Among these health concerns, hematological emergencies remain a critical yet often overlooked aspect of cyclone-related morbidity and mortality. Injuries sustained during cyclones, coupled with environmental and nutritional challenges, create conditions that predispose affected individuals to severe blood-related disorders, including trauma-induced coagulopathy (TIC), acute anemia, disseminated intravascular coagulation (DIC), and sepsis-induced hematological dysfunction. Addressing these complications requires a coordinated approach, with hematologists playing a central role in disaster response and management^[1–7]^. Hematological emergencies in cyclone disasters arise from both direct and indirect causes. Direct causes include trauma from collapsing structures, flying debris, and water-related injuries, leading to significant blood loss and coagulation abnormalities. Indirect causes involve infections due to contaminated water and inadequate sanitation, malnutrition resulting from food shortages, and chronic conditions exacerbated by disrupted healthcare services. These factors contribute to an increased incidence of hematological disorders such as hemolysis, thrombocytopenia, leukocytosis, and thrombotic complications^[8,9]^.

**HIGHLIGHTS**

- Cyclones exacerbate hematological complications, including trauma-induced anemia and coagulopathies.
- Hematologists play a critical role in managing blood loss, coagulation disorders, and infections in disaster settings.
- Disaster preparedness requires efficient blood supply management and advanced diagnostic tools.
- Hematologists collaborate with multidisciplinary teams to improve patient outcomes during emergencies.
- Emerging technologies and strategies, such as telemedicine and portable diagnostics, enhance disaster response and recovery efforts.

One of the primary concerns following a cyclone disaster is TIC, a complex condition characterized by impaired clot formation due to severe blood loss, hypothermia, and acidosis. Without timely intervention, TIC can lead to hemorrhagic shock, multi-organ failure, and death. Hematologists must rapidly assess coagulation status and administer appropriate transfusion therapy, including packed red blood cells (PRBCs), plasma, and clotting factor concentrates. The availability of point-of-care coagulation testing and hemostatic agents plays a crucial role in managing these patients effectively^[10–12]^. Another major hematological complication is acute anemia, which arises from significant blood loss, hemolysis due to infections, and nutritional deficiencies. Cyclone survivors, particularly those in displaced populations, often suffer from iron deficiency anemia due to limited access to food and medical care. Pregnant women, children, and individuals with preexisting hematological conditions are at heightened risk. Addressing anemia in disaster settings requires a strategic approach that includes blood transfusions, iron supplementation, and nutritional interventions^[13,14]^. Infections also contribute significantly to hematological dysfunction in cyclone-affected populations. Contaminated water sources and overcrowded shelters facilitate the spread of bacterial and viral infections, leading to sepsis – a major cause of hematological abnormalities such as thrombocytopenia, leukocytosis, and DIC. Conditions like dengue fever and malaria, which are common in cyclone-prone regions, exacerbate blood-related complications by causing hemolysis and severe thrombocytopenia. Hematologists must be actively involved in monitoring infection-associated hematological changes and guiding appropriate therapeutic interventions^[15,16]^. Beyond immediate disaster response, long-term hematological surveillance is essential to prevent chronic complications arising from cyclone-induced hematological emergencies. Prolonged nutritional deficiencies, untreated anemia, and recurrent infections can lead to long-term hematopoietic dysfunction[17]. Establishing follow-up care programs for affected individuals, particularly vulnerable populations, ensures early detection and management of post-disaster hematological disorders. Public health initiatives that focus on rebuilding healthcare infrastructure and strengthening hematology services in disaster-prone regions are crucial for reducing future morbidity and mortality. Given the complexity of cyclone-induced hematological emergencies, there is an urgent need for a well-structured, interdisciplinary response strategy that includes hematologists, emergency physicians, disaster management teams, and public health officials. By integrating hematology into disaster preparedness plans, ensuring adequate blood bank resources, and establishing emergency transfusion protocols, healthcare systems can significantly improve outcomes for cyclone-affected individuals.

## Aim

The aim of this review is to explore the critical role of hematologists in managing cyclone-induced hematological emergencies, focusing on preparedness, response strategies, and long-term recovery.

## Review methods

In conducting this review on *Cyclone-Induced Hematological Emergencies: Preparedness and Response from a Hematologist’s Viewpoint*, a structured and methodical approach was employed to ensure comprehensive coverage of relevant literature while minimizing potential biases. The review was designed as a narrative synthesis, drawing from diverse sources to integrate current knowledge, practices, and recommendations related to hematological complications in the context of cyclone-induced emergencies.

## Search strategy and databases used

The literature search was carried out across multiple electronic databases, including PubMed, Google Scholar, Scopus, and the World Health Organization Library. The search covered publications from 2000 to 2024, focusing on both peer-reviewed articles and reputable gray literature sources such as disaster response reports, public health bulletins, and guidelines from international agencies like the World Health Organization (WHO), Red Cross, and CDC. The combination of keywords used in the search included: *“cyclone,” “hematological complications,” “disaster medicine,” “coagulopathy,” “dilutional coagulopathy,” “thrombocytopenia,” “disaster preparedness,” “blood disorders in natural disasters,” “emergency hematology,”* and *“point-of-care testing in disaster settings.”* Boolean operators (AND/OR) and truncation techniques were used to refine search results and maximize relevance.

## Inclusion and exclusion criteria

To ensure focus and consistency, the following inclusion criteria were applied:

- Studies or reports discussing hematological conditions directly or indirectly related to cyclones or natural disasters.
- Articles that addressed preparedness strategies, emergency medical response, or diagnostic and therapeutic approaches in disaster-related hematology.
- Publications in English from the year 2000 onward to reflect contemporary practices and recent disaster experiences.
- Peer-reviewed journals, governmental or intergovernmental reports, and emergency response documentation from credible organizations.

Conversely, exclusion criteria comprised:

- Articles unrelated to natural disasters or those focusing solely on earthquakes, tsunamis, or man-made disasters without any overlap with cyclone-related contexts.

Studies that did not discuss hematological parameters or complications in any meaningful capacity.

Non-English publications for which reliable translations could not be sourced.

Articles with inaccessible full texts or those lacking sufficient methodological detail for assessment.

## Techniques to eliminate biases

To reduce selection and interpretation biases, multiple strategies were incorporated. First, the literature screening process was conducted in two phases: initial screening by title and abstract, followed by full-text evaluation. Each article was assessed independently by two reviewers, and discrepancies in article inclusion were resolved through discussion and consensus. A third reviewer was consulted when disagreements persisted. Additionally, an effort was made to include diverse geographic contexts and a mix of high-income and low- and middle-income country data to avoid regional bias. Where multiple sources reported on the same disaster, cross-verification was performed to assess the consistency and reliability of the findings. Grey literature was critically appraised for credibility and relevance, using tools such as the Authority, Accuracy, Coverage, Objectivity, Date, and Significance checklist. Moreover, triangulation of evidence was applied, whereby findings were compared and corroborated across different document types – scientific articles, field reports, and expert opinions – to develop a balanced and well-rounded perspective. Any potential author bias in interpreting the findings was mitigated by maintaining transparency in the review methodology and clearly distinguishing between evidence-based conclusions and commentary.

## Review limitations

While this review aimed to provide a comprehensive exploration of cyclone-induced hematological emergencies from a hematologist’s perspective, certain limitations were encountered that must be acknowledged to contextualize the strength and scope of the conclusions drawn. One of the foremost limitations is the scarcity of specific, high-quality, and systematically collected data on hematological complications directly linked to cyclonic disasters. Much of the available literature is either anecdotal, region-specific, or embedded within broader public health reports, making it difficult to draw generalized inferences or establish prevalence rates of specific conditions such as dilutional coagulopathy, thrombocytopenia, or anemia. The lack of access to a global registry or uniform disaster health surveillance further limited the capacity to present comparative data across different settings or timelines. Additionally, the heterogeneity of the included sources poses a challenge. Publications reviewed included case reports, observational studies, emergency response guidelines, and expert opinion pieces, all of which vary in methodological rigor and depth. Due to the nature of disaster medicine, many reports are written rapidly, with an emphasis on immediate outcomes and logistical response rather than on detailed clinical or hematological analysis. As such, data synthesis was largely narrative rather than quantitative, and the findings should be interpreted as illustrative rather than definitive.

Another key limitation stems from the lack of standardization in laboratory diagnostic approaches reported across various disaster zones. The availability and utilization of laboratory resources, such as point-of-care testing or viscoelastic monitoring (e.g. Rotational Thromboelastometry (ROTEM), Thromboelastography (TEG)), differ significantly between regions, making it difficult to evaluate the consistency of hematological assessment and its influence on patient outcomes. This also constrained the review’s ability to comment on best practices regarding diagnostic strategy in cyclone-induced coagulopathies. Furthermore, language and publication bias may have played a role, as many valuable reports from cyclone-prone regions – particularly in low-resource settings – may not be published in widely indexed journals or may exist only in local languages, limiting their accessibility. Finally, due to the emergent and dynamic nature of cyclones and their aftermath, long-term follow-up data on hematological outcomes are rarely available, thereby limiting the discussion on chronic sequelae or the effectiveness of ongoing interventions.

## Hematological complications in cyclone disasters

Cyclone disasters lead to a wide range of hematological complications, primarily due to traumatic injuries, systemic inflammatory responses, and infections. These complications can significantly impact morbidity and mortality, particularly in vulnerable populations such as children, pregnant women, the elderly, and individuals with preexisting hematological disorders. The interplay of severe blood loss, coagulation abnormalities, and infection-induced hematological dysfunction necessitates prompt and specialized intervention by hematologists^[8,18,19]^.

### TIC and DIC

One of the most immediate hematological challenges following a cyclone is TIC, a life-threatening condition that arises from severe blood loss, hypothermia, acidosis, and systemic inflammation. Cyclone-related injuries such as blunt force trauma, crush injuries, and penetrating wounds disrupt normal coagulation pathways, leading to excessive bleeding and impaired clot formation. The “lethal triad” of hypothermia, acidosis, and coagulopathy further exacerbates hemorrhagic shock, increasing the risk of multi-organ failure. Hematologists play a critical role in assessing coagulation status and administering appropriate blood component therapy, including PRBCs, fresh frozen plasma (FFP), platelets, and clotting factor concentrates[20]. DIC is a severe hematological disorder that can develop in cyclone victims due to sepsis, massive trauma, or systemic inflammatory responses. In this condition, widespread activation of the coagulation system leads to the formation of microthrombi, resulting in organ ischemia, followed by uncontrolled bleeding due to consumption of clotting factors and platelets. DIC is often associated with wound infections, bacterial sepsis from contaminated water sources, and multi-organ dysfunction. Management includes early diagnosis using coagulation profiles (elevated D-dimer, prolonged prothrombin time (PT)/International Normalized Ratio (INR), and thrombocytopenia), supportive care, and administration of clotting factors or anticoagulants, depending on the phase of the disease^[21–23]^.

### Acute anemia and blood loss

Severe blood loss from injuries, coupled with nutritional deficiencies and hemolysis due to infections, contributes to acute anemia in cyclone survivors. Anemia is particularly concerning in displaced populations where access to iron-rich foods and medical care is limited. Pregnant women, children, and individuals with chronic illnesses such as sickle cell disease (SCD) or thalassemia face an increased risk of life-threatening anemia. Timely transfusion support, iron supplementation, and erythropoietin therapy may be required to stabilize affected individuals and prevent long-term complications[21].

### Sepsis-associated hematological dysfunctions

In post-cyclone environments, poor sanitation and limited access to clean water increase the incidence of bacterial, viral, and fungal infections, leading to sepsis-associated hematological complications. Sepsis triggers a systemic inflammatory response that alters hematopoiesis, causing leukocytosis, neutropenia, thrombocytopenia, and hemolysis. Severe sepsis can result in septic shock and multi-organ failure, with profound impacts on the coagulation system. Hematologists must closely monitor white blood cell counts, platelet levels, inflammatory markers (C-Reactive Protein [CRP], procalcitonin), and coagulation profiles to guide early intervention with antibiotics, fluid resuscitation, and supportive hematological therapies[24].

### Vector-borne and waterborne infections affecting hematopoiesis

Cyclone-prone regions often experience outbreaks of vector-borne diseases (dengue fever, malaria, leptospirosis) and waterborne infections (cholera, typhoid, leptospirosis) following a disaster. These infections can directly or indirectly lead to hematological complications:

- Dengue fever causes severe thrombocytopenia, capillary leakage, and hemorrhagic complications, necessitating platelet transfusions and supportive care.
- Malaria leads to hemolytic anemia, thrombocytopenia, and splenic dysfunction, requiring antimalarial therapy and blood transfusions in severe cases.
- Leptospirosis and typhoid fever can cause DIC and hemolysis, further complicating post-cyclone recovery.

Hematologists must be vigilant in recognizing these infections and managing their hematological effects through early diagnosis, appropriate antimicrobial therapy, and blood component replacement as needed[25].

### Thromboembolic risks and immobility-related complications

Cyclone survivors, especially those who are immobilized due to injuries, dehydration, or hospitalization, face an increased risk of venous thromboembolism (VTE), including deep vein thrombosis and pulmonary embolism. The combination of vascular injury, hypercoagulability, and prolonged immobility increases thrombotic risks, particularly in individuals with preexisting conditions such as SCD, cancer, or inherited thrombophilia. Hematologists play a key role in risk assessment, early mobilization strategies, prophylactic anticoagulation, and therapeutic interventions for preventing and managing thrombotic events[26].

### Long-term hematological consequences and recovery challenges

Beyond the acute phase of a cyclone disaster, long-term hematological monitoring is essential to prevent chronic anemia, bone marrow suppression, and immune dysfunction. Survivors, particularly children and pregnant women, may suffer from prolonged iron deficiency, vitamin B12 or folate deficiency, and post-infectious bone marrow suppression. Additionally, individuals with SCD or hemophilia may experience worsening disease symptoms due to inadequate access to specialized care. Strengthening hematology services in disaster-prone areas, establishing follow-up clinics, and implementing nutritional rehabilitation programs are crucial for preventing long-term complications[27].

## Hematologists’ role in disaster preparedness and response

Hematologists play a critical role in both the preparedness for and the response to natural disasters, particularly in managing the hematological complications that arise in the aftermath of such events. Their expertise in diagnosing and treating blood-related disorders is essential in mitigating the morbidity and mortality associated with cyclone-induced injuries, infections, and systemic complications. Disaster preparedness involves not only having the technical knowledge to manage these conditions but also ensuring that adequate resources, protocols, and systems are in place for rapid and effective intervention[28].

### Blood supply and blood bank management

A cornerstone of disaster preparedness for hematologists is ensuring that there is an adequate, well-maintained blood supply in anticipation of natural disasters. This includes the strategic storage of blood components, such as red blood cells (RBCs), plasma, platelets, and clotting factors, which are essential for managing trauma-induced anemia, coagulopathy, and transfusion needs in disaster settings. Hematologists are responsible for blood bank management, ensuring that blood donations are collected, stored, and transported efficiently. They also need to work in coordination with public health agencies to establish emergency blood donation drives, preparing for a surge in demand following a cyclone disaster. Additionally, rapid blood typing and cross-matching capabilities must be in place, especially in regions with high incidences of rare blood groups. Hematologists can help design protocols for safe blood transfusions, ensuring that transfusions are carried out with appropriate screening for infections such as HIV, hepatitis B, and hepatitis C. In a cyclone disaster, when medical facilities are overwhelmed, ensuring that blood banks can rapidly deliver safe, compatible blood is paramount for survival^[21,29–31]^.

### Coagulation and trauma management

One of the most urgent tasks for hematologists during and after a cyclone disaster is the management of TIC, which can occur when individuals suffer from massive blood loss due to injuries. Hematologists must be proficient in using point-of-care coagulation testing to quickly assess the severity of coagulopathies and determine the appropriate transfusion protocols. This includes the administration of FFP, platelets, cryoprecipitate, and clotting factors for patients with bleeding disorders, as well as PRBCs for those with acute blood loss. Hematologists must also be involved in developing strategies for early hemorrhage control, including tourniquets and wound management, until definitive medical care can be provided. Given the potential for mass casualties in cyclone disasters, trauma triage protocols must be in place to prioritize patients based on their hematological status, ensuring that the most critically ill are stabilized first. Hematologists must work in coordination with emergency physicians, surgeons, and anesthesiologists to ensure that patients with severe coagulopathies or blood loss receive timely and adequate care. This collaboration is crucial for improving patient survival rates, particularly in the chaotic and resource-limited conditions that often follow natural disasters^[32–34]^.

### Infection control and hematological monitoring

In the aftermath of a cyclone, infectious diseases such as dengue, malaria, cholera, and typhoid fever can rapidly spread due to poor sanitation and overcrowded shelters. Infections can severely impact the hematological system, leading to thrombocytopenia, hemolysis, sepsis-induced coagulopathy, and DIC. Hematologists must be involved in monitoring hematological parameters such as white blood cell counts, platelet counts, and coagulation profiles to guide the management of these complications. Additionally, antimicrobial stewardship becomes critical in managing sepsis and other infection-related complications. Hematologists can assist in identifying the appropriate antibiotics or antimalarial treatments based on laboratory tests, reducing the risk of secondary infections that complicate blood-related disorders. They must also work closely with infectious disease specialists to monitor patients for signs of sepsis, ensuring that appropriate blood cultures are taken and that appropriate therapeutic interventions are started promptly[35].

### Nutritional support and long-term blood disorder management

The long-term effects of nutritional deficiencies following a cyclone disaster are significant. Limited access to nutritious food and clean water increases the risk of iron deficiency anemia, vitamin B12 deficiency, and folate deficiency, which can complicate recovery. Hematologists have an important role in addressing these nutritional issues by advocating for the provision of fortified foods, iron supplementation, and nutritional support programs aimed at preventing chronic anemia. For individuals with preexisting blood disorders such as SCD, hematologists must provide adequate management to prevent crises exacerbated by nutritional deficiencies and environmental stressors. In addition to nutritional support, rehabilitation programs focusing on the long-term care of hematological complications are essential. Hematologists can help design and implement follow-up care plans for patients who have recovered from acute blood loss or infection but remain at risk for ongoing hematological issues. These plans may include routine blood testing, iron therapy, and monitoring for late-onset complications such as chronic anemia, thrombocytopenia, or splenomegaly[36].

### Training and collaboration with emergency responders

Hematologists play an essential role in training first responders and disaster relief teams on the recognition and management of hematological emergencies. This training includes educating emergency medical personnel about the early signs of blood loss, coagulopathies, and infection-related hematological dysfunctions. Hematologists should also work with disaster management teams to ensure that adequate protocols for blood product storage, transfusion safety, and coagulation management are integrated into the overall disaster response framework. Collaboration with global health organizations and local healthcare providers is vital for ensuring that international aid teams can effectively provide blood products and specialized hematological care. This requires pre-arranged agreements with blood banks, trauma centers, and field hospitals, enabling swift logistical coordination in the event of a disaster[37].

### Psychological support and blood disorder awareness

Finally, the psychological toll of a cyclone disaster – on top of physical injuries and blood disorders – can lead to a heightened risk of psychosomatic hematological disorders such as stress-induced thrombosis or anemia. Hematologists must address the emotional and psychological needs of survivors, helping to build awareness about the physical and mental health challenges of disaster recovery. This can include working with psychologists and social workers to provide a holistic approach to patient care that addresses both the hematological and emotional impacts of disaster survival^[38,39]^.

## Dilutional coagulopathy

Dilutional coagulopathy refers to a condition in which the clotting ability of the blood is impaired due to the excessive dilution of blood components, primarily platelets, clotting factors, and fibrinogen, typically following large-volume fluid resuscitation or massive transfusions. This can lead to abnormal bleeding, especially in the context of trauma and critical illness. In the aftermath of a cyclone, trauma-related injuries, flood-induced infections, and the overall challenge of maintaining homeostasis in a resource-limited setting increase the likelihood of dilutional coagulopathy in affected individuals. Hematologists, with their expertise in coagulation, play a vital role in identifying and managing this condition, which can have severe consequences in emergency settings^[39,40]^.

## Pathophysiology of dilutional coagulopathy in cyclone disasters

In cyclone-induced hematological emergencies, the overwhelming challenge is not only treating trauma-related injuries but also managing the fluid balance in patients. The critical issue arises when large volumes of intravenous fluids are administered to counteract shock or to replace lost blood, leading to a dilution of plasma proteins, clotting factors, and platelets. The sudden loss of these critical components increases the risk of uncontrolled bleeding. In cyclone disasters, where rapid triage is essential and blood products may be limited, dilutional coagulopathy is a frequent complication in trauma patients, especially those undergoing massive transfusion protocols (MTP) or those in need of fluid resuscitation due to flood-related injuries. One of the mechanisms contributing to dilutional coagulopathy is the depletion of clotting factors, as the transfusion of crystalloid or colloid fluids does not replace essential clotting proteins like fibrinogen or factors II, VII, and X. The overuse of fluid resuscitation compounds the problem, as these fluids dilute the natural clotting mechanisms in the body. Additionally, the platelet count may decrease significantly, further impairing coagulation, which can lead to spontaneous bleeding and complications like DIC^[41–44]^.

## Diagnosis and challenges

Diagnosing dilutional coagulopathy in cyclone-induced emergencies is complex, as it is often difficult to perform comprehensive diagnostic tests in a timely manner. Hematologists rely on clinical judgment and available diagnostic tools such as complete blood count, PT, activated partial thromboplastin time, and fibrinogen levels. However, due to the widespread destruction of infrastructure and limited access to laboratories post-cyclone, obtaining these results can be delayed. Thus, the management of dilutional coagulopathy often occurs based on the clinical presentation and immediate response to fluid resuscitation. Early recognition is essential, and signs such as uncontrolled bleeding, hypotension, tachycardia, and anemia should raise suspicion for dilutional coagulopathy. A hematologist’s expertise is crucial in identifying the early markers of coagulopathy even when laboratory results are unavailable, and clinical interventions should be initiated promptly to prevent worsening of the condition^[45,46]^.

## Preparedness and response strategies

In preparing for cyclone-induced hematological emergencies, it is essential to include dilutional coagulopathy in disaster preparedness protocols. Hematologists should advocate for the pre-positioning of blood products such as FFP and cryoprecipitate, which can help replenish coagulation factors in patients who have undergone massive fluid resuscitation or transfusions. Additionally, platelet concentrates should be stocked and available for transfusion when indicated, especially in cases of trauma where platelet counts may be dangerously low. A robust MTP should be in place to guide the management of massive hemorrhage. This protocol should involve the timely administration of a combination of RBCs, platelets, FFP, and cryoprecipitate in appropriate ratios to prevent dilutional coagulopathy. For example, FFP should be given alongside RBCs to replenish clotting factors and maintain a balance in the patient’s coagulation status. The role of the hematologist is pivotal in ensuring that transfusions are performed in a controlled manner, with close monitoring for the development of coagulopathy and ensuring that adequate volumes of blood products are available. In addition to fluid management and transfusion protocols, early goal-directed therapy should be adopted, which includes aggressive monitoring of vital signs, hemodynamic stability, and laboratory tests, as feasible. Given the challenges in delivering comprehensive care post-cyclone, a holistic approach that includes not only the hematological aspects but also the management of trauma, infections, and other organ systems is necessary. Coordination with emergency medicine teams, trauma surgeons, and infectious disease specialists is essential to manage the broad spectrum of complications that arise in such crises^[46,47]^.

## Post-cyclone care and long-term monitoring

After the immediate crisis phase has passed, ongoing care for patients who have experienced dilutional coagulopathy is crucial. Post-cyclone recovery can be prolonged, and patients may face long-term hematological complications such as persistent bleeding, anemia, and chronic coagulopathy. Hematologists must be involved in monitoring coagulation profiles during the recovery phase, particularly in patients who required significant transfusions or fluid resuscitation. Fibrinogen levels, platelet counts, and coagulation factor activity should be regularly assessed to prevent further bleeding episodes or the onset of thrombotic complications. Patients who have suffered dilutional coagulopathy should also be monitored for secondary infections, which can exacerbate hematological conditions. For example, infections such as sepsis can lead to DIC, complicating recovery and requiring further hematological interventions. Hematologists must work alongside other specialties to provide holistic care, ensuring that patients receive comprehensive treatment that includes not only blood component replacement but also the management of underlying infection and organ dysfunction[48].

## Challenges in hematological disaster preparedness and response

Despite the critical role hematologists play in managing cyclone-induced hematological emergencies, several challenges persist in ensuring effective preparedness and response. These challenges span across logistical, infrastructural, and social dimensions, requiring coordinated efforts and long-term planning to address[28].

### Limited resources and infrastructure

In many regions prone to cyclones, especially in low-resource settings, healthcare infrastructure may be insufficient to manage the surge in hematological emergencies. Hospitals and clinics may lack **s**ufficient blood storage facilities, laboratory capacity for point-of-care diagnostics, and trained personnel capable of managing complex hematological disorders. In disaster scenarios, the overburdened healthcare system may also face shortages of blood products such as plasma, platelets, and PRBCs, further compounding the challenges for hematologists trying to stabilize and treat trauma and infection-related blood complications. Moreover, the lack of transportation infrastructure can severely hinder the delivery of critical blood supplies to affected areas. Hematologists may struggle to coordinate with blood banks and donor centers for emergency blood product distribution, which could delay treatment for patients needing transfusions or specialized care. The infrastructure limitations also extend to communication systems, making it difficult to maintain effective coordination among healthcare facilities and emergency teams during a disaster[9].

### Inadequate training and expertise

Another significant challenge is the limited training and expertise of emergency responders, including paramedics, emergency medical technicians, and even some general physicians, in managing hematological emergencies in a disaster context. Many responders are not sufficiently trained in the rapid recognition and treatment of blood disorders such as coagulopathies, anemia, and thrombocytopenia, which are common after traumatic injuries or infections in cyclone disasters. Additionally, the lack of specialized hematologists in some disaster zones can lead to delays in accurate diagnosis and timely treatment, increasing the risk of complications and deaths. To overcome this challenge, it is essential for hematologists to be involved in training programs for emergency medical teams and other healthcare workers, ensuring they understand how to properly manage hematological issues in disaster settings. However, limited access to training resources in disaster-prone regions can make this difficult to achieve[49].

### Psychological and social barriers

The psychological toll of surviving a cyclone disaster is immense and can contribute to significant challenges in treating hematological disorders. Many individuals experience trauma-induced stress and mental health issues such as post-traumatic stress disorder, which can exacerbate or complicate existing blood disorders. These patients may be more prone to stress-induced thrombosis, anemia, or even coagulation problems. However, mental health care often receives less priority during disaster response efforts, limiting the holistic management of individuals affected by blood disorders. Additionally, social stigmas and cultural beliefs about blood disorders can complicate treatment efforts. For instance, individuals with chronic hematological conditions such as SCD or hemophilia may face discrimination or reluctance to seek treatment, particularly if healthcare facilities are overwhelmed and resources are scarce. These social barriers need to be addressed as part of a comprehensive disaster response that incorporates both physical and psychological care[50].

### Challenges in blood product availability and safety

In the aftermath of a cyclone, the demand for blood products skyrockets due to trauma, surgical interventions, and infectious complications. However, there is often a limited supply of blood in disaster-prone regions, making it difficult to meet the urgent needs of disaster victims. The safety of blood products also becomes a critical issue, as inadequate screening procedures or poor storage conditions may lead to contamination, increasing the risk of transfusion-transmitted infections such as HIV, hepatitis, and malaria. Furthermore, rare blood groups pose an additional challenge during blood transfusions in these regions, where there may not be sufficient cross-matching or a database for rare blood type inventories. This issue requires hematologists to plan for alternative blood product sources, such as blood substitutes or cryopreserved blood, but these options are often not readily available or feasible in disaster settings[51].

### Coordination and logistics in crisis situations

Effective coordination between various healthcare providers, local authorities, and humanitarian organizations is essential in responding to the hematological complications of cyclone disasters. However, logistical challenges such as transportation delays, communication breakdowns, and inconsistent supply chains can disrupt timely delivery of critical care. Hematologists often find themselves in a position where they must triage and prioritize treatment, working under extreme pressure to deliver the best care possible with limited resources. The lack of coordination can also hinder access to specialized diagnostic services, making it difficult to accurately assess and treat hematological emergencies[52].

### Delayed or insufficient funding

Hematologists may also face the challenge of insufficient funding for disaster preparedness and response. While many organizations and governments allocate funds for emergency relief, these budgets may not always account for hematological emergencies or blood-related complications, particularly in underdeveloped regions. The delayed allocation of resources can result in a shortage of blood supplies, medications, and diagnostic equipment, severely limiting the ability of hematologists to respond effectively. Early, adequate funding is necessary for setting up blood collection points, purchasing refrigerated storage for blood products, and establishing mobile clinics capable of managing blood disorders in remote areas[53].

## Future perspectives in hematological disaster preparedness and response

As the frequency and intensity of cyclones increase due to climate change, there is an urgent need to enhance hematological disaster preparedness and response strategies. The future of hematological care during such disasters lies in improving systems, technologies, and collaborative efforts to effectively manage hematological complications. Several emerging approaches can help strengthen preparedness, response, and recovery, ensuring that hematologists can more effectively address the complex and urgent needs of cyclone survivors[54].

### Advancements in technology and diagnostics

The future of hematological disaster management will likely see the integration of cutting-edge technologies to improve diagnosis, monitoring, and treatment of hematological conditions. The advent of point-of-care diagnostic tools can enable rapid detection of blood disorders such as coagulopathies, anemia, and thrombocytopenia in the field. For instance, portable hematology analyzers can be used in disaster zones to quickly assess blood cell counts and coagulation parameters, allowing hematologists to make faster decisions and prioritize care. Furthermore, remote monitoring technologies and telemedicine platforms can allow hematologists to consult with local healthcare providers in real time, improving diagnostic accuracy and enabling specialized treatment even in remote areas. Artificial intelligence could also play a crucial role in analyzing patient data quickly to predict potential complications or triage patients based on the severity of their hematological issues. These technologies, combined with mobile blood banks and drone delivery systems, could revolutionize the way blood products are distributed and monitored in the wake of a cyclone disaster[55].

### Enhanced blood donation campaigns and blood supply chain innovations

One of the most pressing concerns in cyclone-induced hematological emergencies is the availability of sufficient blood products. To address this, enhanced blood donation campaigns and innovative blood supply chain models will be necessary. Future strategies may involve community-based blood donation initiatives that encourage local populations to donate blood in preparation for natural disasters. These campaigns could use digital platforms and social media to raise awareness and mobilize donors ahead of storm seasons, ensuring that blood stocks are at optimal levels before disaster strikes. Additionally, advancements in blood product preservation and transportation will be critical. The development of more durable and portable blood storage technologies can ensure that blood products remain viable longer and are easier to transport to remote or hard-hit areas. Freeze-dried blood components or synthetic blood substitutes may also become viable alternatives in the future, providing more flexibility in meeting the emergency needs of disaster-stricken populations[56].

### Strengthening collaborative networks and multidisciplinary teams

In the future, a key area for improvement in disaster response will be the strengthening of collaborative networks and multidisciplinary teams. Hematologists should be integrated into national disaster response plans and global disaster relief networks, ensuring that expertise in blood disorders is readily available when needed. Enhanced cross-border cooperation could facilitate the swift exchange of resources, blood products, and medical personnel between affected regions and unaffected areas. Collaboration between hematologists and other medical specialties, such as trauma surgeons, infectious disease experts, and mental health professionals, will improve the comprehensive care of patients suffering from hematological complications. The future could see the establishment of disaster medicine teams specifically trained in managing hematological emergencies, helping to streamline the response process and reduce delays in care[57].

### Education, training, and capacity building

For future hematological disaster responses to be more effective, there must be a focus on education and capacity building at multiple levels. Hematologists, along with emergency medical teams, will benefit from regular disaster simulation drills to practice managing large-scale hematological emergencies. In addition, training programs should be developed to ensure that local healthcare workers in disaster-prone areas are equipped with the skills to manage hematological complications during cyclones and other natural disasters. Furthermore, the establishment of disaster response educational frameworks could integrate hematological training into medical curricula, preparing the next generation of doctors, nurses, and emergency responders to handle blood-related emergencies effectively. Online platforms and virtual training programs could also make specialized education more accessible to healthcare providers in remote regions[58].

### Long-term research and development in hematological disaster medicine

In the coming years, it is essential to invest in research that explores the long-term health impacts of cyclone-induced hematological emergencies. Studies should focus on the longitudinal effects of traumatic injuries, infections, and stress on blood health, and identify novel therapies to mitigate these impacts. For example, research into hematological biomarkers could lead to new diagnostic tools to predict which individuals are most at risk for blood disorders following disasters. Additionally, the future of disaster medicine may see the development of specialized therapies for hematological conditions exacerbated by disasters. Gene therapies, biologic agents, and new anticoagulants or hemostatic agents could offer more targeted treatments for conditions like coagulopathy and acute anemia in disaster situations. Collaborative partnerships between academic institutions, government agencies, and humanitarian organizations will be essential to drive this research forward and ensure that innovations are integrated into real-world disaster response efforts^[59,60]^.

### Mental health integration in hematological care

As mental health plays an increasingly recognized role in the physical recovery from disasters, the integration of psychological care into hematological disaster management will be a significant focus for the future. Hematologists will need to work alongside mental health professionals to address the psychosomatic and psychological aspects of disaster-related blood disorders. This includes treating trauma-related conditions such as stress-induced anemia, blood pressure abnormalities, and thrombotic events associated with post-traumatic stress. Incorporating mental health care into the overall disaster response will not only improve patient outcomes but will also promote resilience among survivors. Future protocols for cyclone-induced disasters will likely include mental health screenings alongside hematological assessments, allowing for a more comprehensive and holistic approach to patient care[61].

### Policy development and global advocacy

Finally, the future of hematological disaster preparedness lies in the development of policies and global advocacy to ensure that hematology is prioritized in disaster response plans. Governments, international organizations, and healthcare policymakers should work together to create comprehensive disaster preparedness frameworks that incorporate hematological needs. These policies should focus on building resilient healthcare systems capable of addressing both immediate and long-term hematological issues in disaster contexts. Global advocacy efforts can also help raise awareness about the importance of hematological care in disasters and promote the integration of blood products and specialized hematology care into international disaster relief guidelines. As the frequency and severity of cyclones continue to increase, it will be crucial for international organizations, such as the WHO, to support the development of a global response strategy to tackle hematological emergencies in disaster-prone regions[61].

## Conclusion

The management of hematological emergencies in the aftermath of cyclones requires a multifaceted, integrated approach involving advanced technologies, innovative strategies, and multidisciplinary collaboration. Hematologists play a crucial role in identifying and addressing the complex hematological issues arising from natural disasters, particularly those exacerbated by trauma, blood loss, infections, and disruptions in healthcare infrastructure. By enhancing diagnostic capabilities, improving blood product availability, and strengthening emergency preparedness, the healthcare system can better respond to the acute and long-term hematological needs of cyclone survivors. Moreover, as the impact of climate change increases the frequency and severity of cyclones, the need for proactive disaster planning becomes more urgent. The future will see a greater emphasis on education, training, and capacity building, ensuring that local healthcare providers and emergency teams are well-prepared to handle hematological crises. Collaboration among hematologists, policymakers, mental health professionals, and humanitarian organizations will be essential in creating sustainable solutions that not only address immediate needs but also promote long-term recovery.

## Data Availability

Not applicable as this a narrative review.

## Research registration unique identifying number (UIN)

Not applicable as this is a narrative review.

## References

[1] Shultz JM Russell J Espinel Z. Epidemiology of tropical cyclones: the dynamics of disaster, disease, and development. Epidemiol Rev 2005;27:21–35. 15958424 10.1093/epirev/mxi011

[2] Gordon GA Young RR. Natural hazards: hurricanes, cyclones, and typhoons. Encycl Secur Emerg Manage. 2021;2021:676–83.

[3] Paul BK. Human injuries caused by Bangladesh’s cyclone Sidr: an empirical study. Natl Hazards 2010;54:483–95.

[4] Hossain MZ Islam MT Sakai T. Impact of tropical cyclones on rural infrastructures in Bangladesh. Agric Eng Int 2008;2008:1.

[5] Saha CK. Dynamics of disaster-induced risk in southwestern coastal Bangladesh: an analysis on tropical Cyclone Aila 2009. Nat Hazards 2015;75:727–54.

[6] Needham HF Keim BD Sathiaraj D. A review of tropical cyclone-generated storm surges: global data sources, observations, and impacts. Revi Geophys 2015;53:545–91.

[7] Mallick B Ahmed B Vogt J. Living with the risks of cyclone disasters in the south-western coastal region of Bangladesh. Environments 2017;4:13.

[8] Erickson TB Brooks J Nilles EJ. Environmental health effects attributed to toxic and infectious agents following hurricanes, cyclones, flash floods and major hydrometeorological events. J Tox Env Health Part B 2019;22:157–71. 10.1080/10937404.2019.1654422 31437111

[9] World Health Organization. Guidance on ensuring a sufficient supply of safe blood and blood components during emergencies. World Health Org 2023.

[10] Zanza C Romenskaya T Racca F. Severe trauma-induced coagulopathy: molecular mechanisms underlying critical illness. Int J Mol Sci 2023;24:7118. 37108280 10.3390/ijms24087118 PMC10138568

[11] Savioli G Ceresa IF Caneva L. Trauma-induced coagulopathy: overview of an emerging medical problem from pathophysiology to outcomes. Medicines 2021;8:16. 33805197 10.3390/medicines8040016 PMC8064317

[12] Barrett L Curry N Abu-Hanna J. Experimental models of traumatic injuries: do they capture the coagulopathy and underlying endotheliopathy induced by human trauma? Int J Mol Sci 2023;24:11174. 37446351 10.3390/ijms241311174 PMC10343021

[13] Robertson JJ Brem E Koyfman A. The acute hemolytic anemias: the importance of emergency diagnosis and management. J Emerg Med 2017;53:202–11. 28408234 10.1016/j.jemermed.2017.02.018

[14] Munro N. Hematologic complications of critical illness: anemia, neutropenia, thrombocytopenia, and more. AACN Adv Crit Care 2009;20:145–54. 19411872 10.1097/NCI.0b013e3181a0d6ea

[15] Parks RM Benavides J Anderson GB. Association of tropical cyclones with county-level mortality in the US. JAMA 2022;327:946–55. 35258534 10.1001/jama.2022.1682 PMC8905400

[16] Wahid SS Raza WA Mahmud I. Climate-related shocks and other stressors associated with depression and anxiety in Bangladesh: a nationally representative panel study. Lancet Planet Health 2023;7:e137–146. 36754469 10.1016/S2542-5196(22)00315-1

[17] Thachil J Owusu-Ofori S Bates I. Haematological diseases in the tropics. Manson’s Trop Infect Dis. 2013;2013:894.

[18] Saulnier DD Ribacke KB von Schreeb J. No calm after the storm: a systematic review of human health following flood and storm disasters. Prehosp Disaster Med 2017;32:568–79. 28606191 10.1017/S1049023X17006574

[19] Calgaro S Borellini M Seni AH. Neonatal intensive care unit evacuation and care during a natural disaster: the experience of cyclone Idai in Beira, Mozambique. Front Pediatr 2020;8:584281. 33194918 10.3389/fped.2020.584281 PMC7642448

[20] McCunn M Ahmed MI Kuza CM. Cutting-edge trauma and emergency care, an issue of anesthesiology clinics. Elsevier Health Sci 2019. 10.1016/j.anclin.2018.11.001 30711238

[21] Gammon RR Rosenbaum L Cooke R. Maintaining adequate donations and a sustainable blood supply: lessons learned. Transfusion 2021;61:294–302. 33206404 10.1111/trf.16145 PMC7753343

[22] Hua-Qiang L Xi-Kang L. Management of multiple injuries in a disaster. Prehosp Disaster Med 2002;17:S80–S80.

[23] Maxatadze D Metreveli Z Chixradze K. One year Georgian experience of nitrous oxide usage in prehospital care and analgesia during transportation. Prehosp Disaster Med 2002;17:S80–S81.

[24] Haraguchi Y Tomoyasu Y Nishi H. Intensive care and disaster medicine: the role of a compendium. Crit Care 2013;17:P279.

[25] Polgreen PM Polgreen EL. Emerging and re-emerging pathogens and diseases, and health consequences of a changing climate. Infect Dis (Auckl). 2016;2016:40.

[26] Sunohara D Miura T Komatsu T. Relationship between the flood disaster caused by the Reiwa first year east Japan typhoon and cardiovascular and cerebrovascular events in Nagano City: the SAVE trial. J Cardiol 2021;78:447–55. 34183228 10.1016/j.jjcc.2021.06.003

[27] Bandaru S Sano S Shimizu Y. Impact of heavy rains of 2018 in western Japan: disaster-induced health outcomes among the population of Innoshima Island. Heliyon 2020;6:1. 10.1016/j.heliyon.2020.e03942 PMC7256463 32490225

[28] Curtis CM Kost GJ Louie RF. Point-of-care hematology and coagulation testing in primary, rural emergency, and disaster care scenarios. Point Care 2012;11:140–45. 23843728 10.1097/POC.0b013e31825a9d3a PMC3703674

[29] Gschwender AN Gillard L. Disaster preparedness in the blood bank. Am Soc Clin Lab Sci 2017;30:250–57.

[30] Kuruppu KK. Management of blood system in disasters. Biologicals 2010;38:87–90. 20149686 10.1016/j.biologicals.2009.10.005

[31] Shander A Goobie SM Warner MA. Essential role of patient blood management in a pandemic: a call for action. Anesth Analg 2020;131:74–85. 32243296 10.1213/ANE.0000000000004844 PMC7173035

[32] Stanworth SJ Dowling K Curry N. Transfusion task force of the British Society for Haematology. Haematological management of major haemorrhage: a British Society for Haematology Guideline. Br J Haematol 2022;198:654–67. 35687716 10.1111/bjh.18275

[33] Rossaint R Afshari A Bouillon B. The European guideline on management of major bleeding and coagulopathy following trauma. Crit Care 2023;27:80. 36859355 10.1186/s13054-023-04327-7 PMC9977110

[34] Spahn DR Bouillon B Cerny V. Management of bleeding and coagulopathy following major trauma: an updated European guideline. Crit Care 2013;17:1–45. 10.1186/cc12685 PMC4056078 23601765

[35] Dainiak N Waselenko JK Armitage JO. The hematologist and radiation casualties. ASH Educ Program Book 2003;2003:473–96. 10.1182/asheducation-2003.1.473 14633795

[36] Greenberg PL Gordeuk V Issaragrisil S. Major hematologic diseases in the developing world—new aspects of diagnosis and management of thalassemia, malarial anemia, and acute leukemia. ASH Educ Program Book 2001;2001:479–98. 10.1182/asheducation-2001.1.479 11723000

[37] Doughty H Chowdhury F Ameh V. Emergency preparedness, resilience and response guidance for UK hospital transfusion teams. Transfus Med 2020;30:177–85. 32020684 10.1111/tme.12665 PMC7317494

[38] Glynn SA Busch MP Schreiber GB; NHLBI REDS Study Group, NHLBI REDS Study Group. Effect of a national disaster on blood supply and safety: the September 11 experience. JAMA. 2003;289:2246–53. 12734136 10.1001/jama.289.17.2246

[39] Uzun S Ediz Ç Mohammadnezhad M. Hematology patients’ metaphorical perceptions of the disease and psychosocial support needs in the treatment process: a phenomenological study from a rural region of Türkiye. Supportive Care Cancer 2025;33:222. 10.1007/s00520-025-09278-z PMC11860990 40000464

[40] Jones DG Nantais J Rezende-Neto JB. Crystalloid resuscitation in trauma patients: deleterious effect of 5L or more in the first 24h. BMC Surg 2018;18:93. 30400852 10.1186/s12893-018-0427-y PMC6219036

[41] Fries D Streif W Haas T. Dilutional coagulopathy, an underestimated problem? Transfus Med Hemother 2004;31:237–42. 10.1055/s-2004-825912 15605299

[42] Hirshberg A Dugas M Banez EI. Minimizing dilutional coagulopathy in exsanguinating hemorrhage: a computer simulation. J Trauma Acute Care Surg 2003;54:454–63. 10.1097/01.TA.0000053245.08642.1F 12634523

[43] Eriksen CF Tønnesen E Ingerslev J. Mechanisms of hydroxyethyl starch-induced dilutional coagulopathy. J Thromb Haemost 2009;7:1099–105. 19422451 10.1111/j.1538-7836.2009.03460.x

[44] Fries D Krismer A Klingler A. Effect of fibrinogen on reversal of dilutional coagulopathy: a porcine model. Br J Anaesth 2005;95:172–77. 15923269 10.1093/bja/aei160

[45] Weiss G Lison S Spannagl M. Expressiveness of global coagulation parameters in dilutional coagulopathy. Br J Anaesth 2010;105:429–36. 20693180 10.1093/bja/aeq199

[46] Haas T Mauch J Weiss M. Management of dilutional coagulopathy during pediatric major surgery. Transfus Med Hemother 2012;39:114–19. 22670129 10.1159/000337245 PMC3364035

[47] Fries D Innerhofer P Reif C. The effect of fibrinogen substitution on reversal of dilutional coagulopathy: an in vitro model. Anesth Analg 2006;102:347–51. 16428520 10.1213/01.ane.0000194359.06286.d4

[48] Schols SE Heemskerk JW van Pampus EC. Correction of coagulation in dilutional coagulopathy: use of kinetic and capacitive coagulation assays to improve hemostasis. Transfus Med Rev 2010;24:44–52. 19962574 10.1016/j.tmrv.2009.09.004

[49] Bloch EM Vermeulen M Murphy E. Blood transfusion safety in Africa: a literature review of infectious disease and organizational challenges. Transfus Med Rev 2012;26:164–80. 21872426 10.1016/j.tmrv.2011.07.006 PMC3668661

[50] Nichols WL Hultin MB James AH. Von Willebrand disease (VWD): evidence-based diagnosis and management guidelines, the National Heart, Lung, and Blood Institute (NHLBI) expert panel report (USA). Haemophilia 2008;14:171–232. 18315614 10.1111/j.1365-2516.2007.01643.x

[51] Farrokhizadeh E Seyfi-Shishavan SA Satoglu SI. Blood supply planning during natural disasters under uncertainty: a novel bi-objective model and an application for red crescent. Ann Oper Res 2022;319:73–113.

[52] Salah S Blood banks and their effectiveness in addressing disasters: A case of the Kenyatta National Hospital (Doctoral dissertation, University of Nairobi). 2014.

[53] Wahyuni RD Pasinringi SA Palutturi S. Hospital laboratory management in dealing with disaster. Gac Sanit. 2021;35:S180–182. 34929806 10.1016/j.gaceta.2021.10.020

[54] Winters TA Cassatt DR Harrison-Peters JR. Considerations of medical preparedness to assess and treat various populations during a radiation public health emergency. Radiat Res 2023;199:301–18. 36656560 10.1667/RADE-22-00148.1 PMC10120400

[55] Jayaram A Dutta R Kim EK. Alternative strategies for emergency blood transfusion in low-resource settings: a scoping review. Transfusion 2024;64:1350–61. 38742837 10.1111/trf.17838

[56] Alshammari HS Alshammari AF Alshammari BF. Multidisciplinary collaboration in emergency medical services. J Positive Psychol Wellbeing 2022;6:695–721.

[57] Gomez D Haas B Ahmed N. Disaster preparedness of Canadian trauma centres: the perspective of medical directors of trauma. Can J Surg 2011;54:9. 21251427 10.1503/cjs.022909 PMC3038366

[58] Obeagu EI Obeagu GU. Mental health and psychosocial effects of natural disaster on HIV Patients. Sciences 2024;4:38–44.

[59] Obeagu EI Obeagu GU. Implications of climatic change on sickle cell anemia: a review. Medicine (Baltimore) 2024;103:e37127. 38335412 10.1097/MD.0000000000037127 PMC10860944

[60] Nwankwo CU Egbule EO Ede KR. Evaluating the impact of rational emotive behavior therapy on post-traumatic stress in farmers with experiences of natural disaster. Medicine (Baltimore) 2024;103:e40244. 39470476 10.1097/MD.0000000000040244 PMC11520993

[61] Obeagu EI Obeagu GU. Adapting to the shifting landscape: implications of climate change for malaria control: a review. Medicine (Baltimore) 2024;103:e39010. 39029063 10.1097/MD.0000000000039010 PMC11398779

